# What Do Adolescents and Young Adults Think a Cigarillo Is? Implications for Health Communication

**DOI:** 10.3390/ijerph18063311

**Published:** 2021-03-23

**Authors:** Jennifer Cornacchione Ross, David M. Reboussin, Seth M. Noar, Kimberly D. Wiseman, Erin L. Sutfin

**Affiliations:** 1 Department of Social Sciences and Health Policy, Wake Forest School of Medicine, Winston Salem, NC 27157, USA; kwiseman@wakehealth.edu (K.D.W.); esutfin@wakehealth.edu (E.L.S.); 2 Department of Biostatistics and Data Science, Wake Forest School of Medicine, Winston Salem, NC 27157, USA; drebouss@wakehealth.edu; 3 Hussman School of Journalism and Media, University of North Carolina, Chapel Hill, NC 27599, USA; noar@unc.edu; 4 Lineberger Comprehensive Cancer Center, University of North Carolina, Chapel Hill, NC 27599, USA

**Keywords:** cigarillo, tobacco, survey, youth, young adults, health communication

## Abstract

Cigarillo use has increased among adolescents and young adults and has remained high. Public education efforts are needed to communicate with these populations about cigarillo use risks, but little is known about the implications of using the term “cigarillo” in such efforts. The study goal was to assess adolescent and young adult perceptions of the term “cigarillo”. We conducted a nationally representative online survey of 3517 adolescents and young adults (ages 13–25). We asked participants “what is a cigarillo?” with several response options. Participants were 49.6% female, 69.8% white, 5.2% reported past 30-day cigarillo use, and 11.6% reported lifetime cigarillo use. The most common response to the question “what is a cigarillo” was “I don’t know” (51% of participants), followed by “a thinner and smaller version of a traditional cigar” (30.1% of participants), which was chosen by 19.4% of adolescents and 36.8% of young adults. Among past 30-day cigarillo users, the most common response was “I don’t know” (54.9%) followed by “a thinner and smaller version of a traditional cigar” (45.1%). Cigarillo users were more likely to select the “a thinner and smaller version of a traditional cigar” response than nonusers. Findings suggest that many adolescents and young adults have varied understandings of the term “cigarillo”. Researchers and practitioners need to ensure that terminology used in health communication campaigns is clearly understood by the target audience to maximize effectiveness.

## 1. Introduction

Cigarillos, a type of cigar product, pose a public health threat because their use causes significant health consequences, including addiction and several types of cancers [1]. Moreover, there are misperceptions about their risks, such as believing they are not addictive [2,3]. In the United States, cigar use among adolescents and young adults remains high, with 14.4% of adolescents (middle and high school students) and 44.5% of young adults (ages 18–24) reporting ever using a cigar [4,5]. In fact, cigars were the second most common tobacco product used by adolescents in the United States in 2019 behind e-cigarettes, with more high school students reporting current use of cigars (7.6%) than cigarettes (5.8%) [5]. Cigarillos are the most commonly used cigar product among adolescents and young adults [4,6]. 

Cigarillos are under the regulatory authority of the Food and Drug Administration (FDA), so it is under its purview to communicate cigarillo health risks to the public [7], which can occur through public education campaigns (e.g., communication campaigns). Substantial evidence exists that communication campaigns are effective at educating the public about the risks of cigarettes in ways that discourage use [8]. However, there is limited research on the effectiveness of messaging for cigarillos [9,10]. Unlike messaging about cigarette smoking, one challenge of messaging for cigar products is the variety of terminology and language used to describe and define these products [11]. Research has demonstrated that including cigarillo brand names (e.g., Swisher Sweets) in survey items improves measurement accuracy [12]. Furthermore, consumers call cigarillos a variety of different names, including brand names [11]. However, inclusion of brand names is not feasible or advisable for larger public communication efforts, which do not want to promote cigarillo brand names or provide smoking cues [13]. It is important to understand how adolescents and young adults understand the term “cigarillo” to inform use of that term in campaigns. This study’s purpose was to assess adolescents’ and young adults’ perceptions of the term “cigarillo” to inform how researchers and health organizations might communicate about cigarillos to the public.

## 2. Materials and Methods

### 2.1. Sample

Data were collected from a nationally representative sample of adolescents and young adults aged 13–25 from March to April 2016. GfK Custom Research administered the survey through their online KnowledgePanel^®^. KnowledgePanel is a probability-based web panel that is representative of the United States, and members are recruited through address-based sampling, which is probability-based sampling of all U.S. addresses from the Delivery Sequence File of the United States Postal Service. Potential participants are invited via an initial invitation letter. For those interested in participating in the panel but who do not have internet access, they are provided with a web-enabled device to complete surveys.

For the current study, GfK contacted KnowledgePanel members between the ages of 18 and 25 years old and invited them to participate in the survey. To recruit 13–17-year-olds and additional 18–25-year-olds, GfK contacted pre-identified adult KnowledgePanel members who had previously reported having a 13–25-year-old in their household and randomly selected a 13–25-year-old from the household to invite to participate in the survey. Consent (participants aged 18–25) and assent (participants aged 13–17) were obtained for each participant, and parental consent was obtained for adolescents aged 13–17. Of 8665 individuals invited to participate in the survey, 52% completed the screener survey; 572 were not eligible (pre-identified adults who stated they did not have a 13–25-year-old living in their household at least 50% of the time or were not a parent or legal guardian) and 289 did not provide consent or assent. The final sample size of completed surveys was 3517 (96.5% survey completion rate by eligible individuals). The Wake Forest University Health Sciences Institutional Review Board approved the study.

### 2.2. Measures

Cigarillo terminology perception. Preceding the section that assessed cigarillo use, participants were asked the question “What is a cigarillo?” with the following response options: the same as a regular cigarette; a cigarette with flavoring (e.g., grape, cherry); the same as a traditional cigar; a thinner and smaller version of a traditional cigar; a cigar used only to smoke marijuana; and I don’t know. We identified a “thinner and smaller version of a traditional cigar” to be the most closely aligned definition from reviewing the legal definition of cigars, literature on cigar product types, and population-based surveys (e.g., Population Assessment of Tobacco and Health) [11,14]. The first two response options (cigarette and cigarette with flavoring) were created to identify whether participants think a cigarillo is a cigarette or a little cigar. The third response option (the same as a traditional cigar) was created to represent large, premium cigars. The marijuana response option (a cigar used only to smoke marijuana) was created because some cigarillo users take out the tobacco and put marijuana into the wrapper. We included the last response option (I don’t know) to allow for participants to state that they did not know the definition rather than guessing.

Cigarillo use behaviors. After answering the cigarillo terminology perception question and moving to the next survey page, participants were provided with the definition of a cigarillo and an image of cigarillos, and cigarillo use behaviors were assessed. Participants were then categorized into one of four mutually exclusive groups. They were classified as past 30-day users if they said they had used a cigarillo on at least one day in the past 30 days. Participants were classified as ever users if they had ever used a cigarillo, even just one time, but not in the past 30 days. Susceptible nonusers were those who had never used a cigarillo and responded with anything other than “definitely not” to the item “If one of your best friends were to offer you a cigarillo, would you use it?”, one item adapted from the Pierce susceptibility scale, which predicts adolescent cigarette smoking initiation [15]. Participants were classified as non-susceptible nonusers if they had never used a cigarillo and were not susceptible (i.e., answered “definitely not” to the susceptibility item).

Demographics. We assessed age (adolescents aged 13–17, young adults aged 18–25), race (white, Black, other), sex (male, female), sexual orientation for young adults and attraction for adolescents, and education as a marker for socioeconomic status (own education for young adults, mother’s education for adolescents).

### 2.3. Analyses

Weights to adjust analyses of the nationally representative sample were created using data from the U.S. Census Bureau Current Population Survey. Estimated proportions and tests were calculated using SURVEYFREQ, SURVEY MEANS, SURVEYREG, and SURVEYLOGISTIC procedures in SAS version 9.4. Response options for “what is a cigarillo?” were categorized into one of three categories for these analyses: (1) “I don’t know”, (2) “a thinner and smaller version of a traditional cigar”, (3) any other response option. Logistic regression analyses were conducted to assess the probability of each user group to select each of these categories of response options.

## 3. Results

Adolescents and young adults in the target population were 49.6% female, 69.8% white, and 22.0% Hispanic, with a mean age of 19.1. Weighted prevalence of cigarillo use status was 5.2% past 30-day users (2.6% adolescents, 6.8% young adults), 11.6% ever (not past 30-day) users (2.6% adolescents, 17.2% young adults), 13.7% susceptible nonusers (14.2% adolescents, 13.4% young adults), and 69.6% non-susceptible nonusers (80.6% adolescents, 62.6% young adults). See Table 1 for sample demographics.

### 3.1. Descriptive Data

When asked what a cigarillo is, the most commonly selected response option among the full sample of adolescents and young adults was “I don’t know” (51.0%), followed by the “a thinner and smaller version of a traditional cigar” (30.1%) (Table 2).

We also assessed responses by cigarillo user status. As a reminder, cigarillo use behaviors were asked of participants after responding to the “what is a cigarillo” item. Among past 30-day cigarillo users, 44.8% selected the “a thinner and smaller version of a traditional cigar”, and 46.3% selected another response option, including “a cigarette with flavoring” (26.4%) and “a cigar used only to smoke marijuana” (14.9%). Only 8.6% of past 30-day cigarillo users selected “I don’t know”. Among ever users, the most common response option was “a thinner and smaller version of a traditional cigar” (54.1%) followed by “I don’t know” (20.2%) and “a cigarette with flavoring” (18.4%). Among susceptible nonusers, 45.9% selected “I don’t know” and 34.1% selected the “a thinner and smaller version of a traditional cigar”. About one-tenth (10.2%) selected “a cigarette with flavoring”. A similar pattern of results was found for non-susceptible nonusers, with 60.3% selecting “I don’t know” and 24.4% selecting “a thinner and smaller version of a traditional cigar” (Table 2). 

We also examined responses by age group. For adolescents, the majority of participants selected “I don’t know” (64.8%) followed by the “a thinner and smaller version of a traditional cigar” (19.4%). Similarly, many young adults responded with “I don’t know” (42.2%), while 36.8% selected “a thinner and smaller version of a traditional cigar”, and 9.8% selected “a cigarette with flavoring” (Table 2).

### 3.2. Group Differences

We examined differences in responses (three categories: “a thinner and smaller version of a traditional cigar”, “I don’t know”, and any other response) across groups. Adolescents were significantly more likely to select a response other than “a thinner and smaller version of a traditional cigar” compared to young adults, *F* (1, 3515) = 79, *p* < 0.001. Furthermore, significant differences in perceptions emerged between user groups, F (3, 3507) = 31, *p* < 0.001. Past 30-day users were not significantly different from ever users or susceptible nonusers in selecting “a thinner and smaller version of a traditional cigar”, but they were significantly more likely to select it compared to non-susceptible nonusers, t (3509) = 4.2, *p* < 0.001. Ever users were more likely to perceive a cigarillo to be “a thinner and smaller version of a traditional cigar” compared to susceptible nonusers (t (3509) = 4.5, *p* < 0.001) and non-susceptible nonusers (t (3509) = 8.9, *p* < 0.001). Non-susceptible nonusers were significantly less likely than susceptible nonusers to perceive cigarillos as “a thinner and smaller version of a traditional cigar”, t (3509) = −3.5, *p* < 0.001.

## 4. Discussion

The goal of this study was to understand adolescents’ and young adults’ perceptions of the term “cigarillo”, which has implications for communicating to these priority populations about the risks of cigarillo use. This study found that there is a general lack of understanding about the term “cigarillo” among adolescents and young adults, which suggests that future campaigns intended to target cigarillo use in this group need to be clear about what a cigarillo is. Although the majority of participants selected “I don’t know” when asked “what is a cigarillo?”, we also found differences in perceptions of the term “cigarillo” based on age group and cigarillo user status.

Young adults selected “a thinner and smaller version of a traditional cigar”, which most closely aligns with what would be considered a “correct” response at a greater frequency than adolescents (36.8% vs. 19.4%). Additionally, those who were users (past 30-day and ever) of cigarillos generally responded more frequently than nonusers with “a thinner and smaller version of a traditional cigar”. Nevertheless, almost half of users still selected another response or reported that they did not know what a cigarillo is. These findings suggest that communication campaigns using the term “cigarillo” are likely to be better understood when targeting users of the product as compared to nonusers. It is important to also effectively communicate cigarillo risks to nonusers to prevent initiation, however. Some past 30-day users may also use cigarillos to smoke marijuana (i.e., blunts) [16], which may help to explain why 15% selected “A cigar used only to smoke marijuana” as their response. Interestingly, though, 28.2% of past 30-day users and 21.5% of ever users reported thinking a cigarillo was a cigarette, selecting “the same as a regular cigarette” or “a cigarette with flavoring”. This finding could possibly be a result of a lack of understanding of or the variety of language used to describe the multiple cigar products on the market, including large premium cigars, little cigars, and cigarillos, and research has found that consumers often perceive little cigars to be the same product as cigarettes [11,17,18].

Our findings suggest that targeting nonusers for prevention of cigarillo uptake may be challenging. Less than 20% of adolescents reported “a thinner and smaller version of a traditional cigar” (the definition that most aligns with the “correct” response) and between 46% and 60% of nonusers (susceptible and non-susceptible) selected “I don’t know”. When developing and implementing communication campaigns aimed at preventing cigarillo use among adolescents and young adults, it will be a challenge to identify how to most effectively communicate the risks of cigarillos given the myriad of ways that people understand and refer to cigarillos [11]. 

One possibility to solve this problem is creating a campaign that educates young people about what cigarillos are, and why they are just as dangerous as cigarettes. Since flavored products are sometimes viewed as less harmful by young people [19], such a campaign could take the opportunity to educate youth about multiple aspects of cigarillos and their risks. Although including brand names and imagery is known to increase the accuracy of prevalence estimates in research [12], including brand names is not likely to be a good practice in communication campaigns. Adding product imagery without branding information may be one solution, but the presence of smoking cues, such as an image of a cigarillo, particularly for tobacco users, may result in increased cravings to smoke [13,20]. Furthermore, including brand names could pose legal challenges. Overall, research is needed to identify whether product imagery increases consumers’ ability to identify the product being communicated about, and whether imagery increases positive perceptions and interest in the product, an unintended consequence of including product imagery. 

Another area for future research is to better understand the causes of these perceptions of the definition of a “cigarillo” and why they vary among adolescents and young adults. For example, what led some people to believe a cigarillo is essentially a cigarette, whereas others view it as only a cigar to smoke marijuana? The ways in which cigarillos are used or perceived to be used may also influence perceptions about their definition [21,22,23,24]. For example, those who use cigarillos exclusively to smoke marijuana (i.e., blunts) may be more likely to define cigarillos as “a cigar used only to smoke marijuana”. However, our study was not designed to assess causes of “cigarillo” term perceptions. A greater understanding can also help to inform communication efforts to improve knowledge and perceptions about cigarillos. This could begin with qualitative work to understand what underpins how young people perceive various types of combustible tobacco products for developing effective messages, extending upon previous work looking at product terminology [11]. Although “cigarillo” is used by researchers and policymakers, it has not necessarily been widely used in public with consumers, which could contribute to participants’ lack of consensus regarding the definition of the term “cigarillo”. Our findings support previous work that has demonstrated a wide range of perceptions about cigar product terminology, including cigarillos [11,17].

These findings should be interpreted with limitations in mind. This study only asked one item about the term “cigarillo” and did not assess the word or product within the context of an actual message or communication campaign, nor were any images of cigarillos or brands shown. It is possible that even though the participants had multiple perceptions of the term “cigarillo”, they could identify the product or know what it is in the context of a communication campaign. Future research should examine this in the broader context of how to most effectively communicate about the harms of cigarillos to young people. Additionally, because there were few adolescent cigarillo users in the sample, we were unable to conduct statistical analyses while stratifying results on both age group and cigarillo user status. However, our findings still provide evidence on understanding and perceptions of the term “cigarillo”. Finally, a significant strength of GfK is that it uses probability-based sampling.

## 5. Conclusions

The goal of this study was to understand how adolescents and young adults perceive the term “cigarillo” to inform future communication efforts aimed at discouraging use. The majority of adolescents and young adults selected “I don’t know” when asked “what is a cigarillo?”. However, differences were identified based on cigarillo user status and age group, demonstrating that using the term “cigarillo” may be better understood in efforts aimed at users. Research should continue to explore the use of the term “cigarillo” or other effective language within the context of communication campaigns aimed at informing adolescents and young adults about the harms of cigarillo use to prevent initiation and reduce use.

## Figures and Tables

**Table 1:** Demographics and cigarillo user status.

Variable	Full Sample N = 3517 N (%) or M ± SE	Adolescents N = 1298 N (%) or M ± SE	Young Adults N = 2219 N (%) or M ± SE
Sex			
Male	1587 (50.4%)	675 (51.1%)	912 (49.9%)
Female	1930 (49.6%)	623 (48.9%)	1307 (50.1%)
Age	19.1 ± 0.07	15.0 ± 0.04	21.6 ± 0.07
Race			
White	2586 (69.8%)	964 (70.7%)	1622 (69.2%)
Black	243 (15.3%)	392 (15.3%)	149 (15.2%)
Other	323 (15.5%)	507 (15.0%)	184 (14.1%)
Hispanic	669 (22.0%)	231 (22.7%)	438 (21.6%)
Sexual Orientation/Attraction			
Heterosexual	3150 (93.9%)	1137 (92.8%)	2013 (94.6%)
Gay, lesbian, or bisexual	239 (6.1%)	88 (7.2%)	151 (5.4%)
Education (mothers for adolescents; self for young adults)			
High school or less	860 (38.9%)	285 (29.8%)	575 (44.6%)
Some college, no degree	1418 (34.0%)	453 (31.6%)	965 (35.4%)
At least a college degree	1235 (27.1%)	556 (38.6%)	679 (19.9%)
Past 30-day cigarillo user	151 (5.2%)	27 (2.6%)	124 (6.8%)
Ever cigarillo user (not past 30 day)	438 (11.6%)	33 (2.6%)	405 (17.2%)
Susceptible cigarillo nonuser	459 (13.7%)	182 (14.2%)	277 (13.4%)
Non-susceptible cigarillo nonusers	2463 (69.6%)	1056 (80.6%)	1407 (62.6%)

Percentages are weighted. Other race includes American Indian/Alaska Native, Asian Indian, Chinese, Filipino, Japanese, Korean, Vietnamese, Native Hawaiian, Pacific Islander, or “other”. Six participants did not respond to the cigarillo use behavior assessment.

**Table 2:** Cigarillo definition perceptions by user status and age group.

A Cigarillo Is…	Full Sample (n = 3517)	Past 30-Day Users (n = 151)	Ever Users (n = 438)	Susceptible Nonusers (n = 459)	Non-Susceptible Nonusers (n = 2463)	Adolescents (N = 1298)	Young Adults (N = 2219)
A thinner & smaller version of a traditional cigar	30.1%	44.8%	54.1%	34.1%	24.4%	19.4%	36.8%
The same as a regular cigarette	5.8%	1.8%	3.1%	5.3%	6.7%	6.8%	5.2%
A cigarette with flavoring (e.g., grape, cherry)	8.4%	26.4%	18.4%	10.2%	5.0%	6.1%	9.8%
The same as a traditional cigar	1.7%	3.2%	1.1%	1.8%	1.7%	1.0%	2.2%
A cigar used only to smoke marijuana	2.2%	14.9%	3.1%	1.9%	1.1%	1.3%	2.7%
I don’t know	51.0%	8.6%	20.2%	45.9%	60.3%	64.8%	42.2%

Percentages are weighted.

## Data Availability

Data are not available at this time due to ongoing analysis. After all study analyses are complete, de-identified datasets will be available upon request.

## References

[1] Chang C.M., Corey C.G., Rostron B.L., Apelberg B.J. (2015). Systematic Review of Cigar Smoking and All Cause and Smoking Related Mortality. BMC Public Health.

[2] Cornacchione J., Wagoner K.G., Wiseman K.D., Kelley D., Noar S.M., Smith M.H., Sutfin E.L. (2016). Adolescent and Young Adult Perceptions of Hookah and Little Cigars/Cigarillos: Implications for Risk Messages. J. Health Commun..

[3] Nyman A.L., Sterling K.L., Weaver S.R., Majeed B.A., Eriksen M.P. (2016). Little Cigars and Cigarillos: Users, Perceptions, and Reasons for Use. Tob. Regul. Sci..

[4] Kasza K.A., Ambrose B.K., Conway K.P., Borek N., Taylor K., Goniewicz M.L., Cummings K.M., Sharma E., Pearson J.L., Green V.R. (2017). Tobacco-Product Use by Adults and Youths in the United States in 2013 and 2014. N. Engl. J. Med..

[5] Wang T.W., Gentzke A.S., Creamer M.R., Cullen K.A., Holder-Hayes E., Sawdey M.D., Anic G.M., Portnoy D.B., Hu S., Homa D.M. (2019). Tobacco Product Use and Associated Factors Among Middle and High School Students—United States, 2019. MMWR Surveill. Summ..

[6] Corey C.G., Holder-Hayes E., Nguyen A.B., Delnevo C.D., Rostron B.L., Bansal-Travers M., Kimmel H.L., Koblitz A., Lambert E., Pearson J.L. (2018). US Adult Cigar Smoking Patterns, Purchasing Behaviors, and Reasons for Use According to Cigar Type: Findings From the Population Assessment of Tobacco and Health (PATH) Study, 2013–2014. Nicotine Tob. Res..

[7] Food and Drug Administration (2016). HHS Deeming Tobacco Products to Be Subject to the Federal Food, Drug, and Cosmetic Act, as Amended by the Family Smoking Prevention and Tobacco Control Act; Restrictions on the Sale and Distribution of Tobacco Products and Required Warning Statements for Tobacco Products. Final Rule. Fed. Regist..

[8] Farrelly M.C., Nonnemaker J., Davis K.C., Hussin A. (2009). The Influence of the National Truth® Campaign on Smoking Initiation. Am. J. Prev. Med..

[9] Kong G., Creamer M.R., Simon P., Cavallo D.A., Ross J.C., Hinds J.T., Fishbein H., Gutierrez K. (2019). Systematic Review of Cigars, Cigarillos, and Little Cigars among Adolescents: Setting Research Agenda to Inform Tobacco Control Policy. Addict. Behav..

[10] Cornacchione Ross J., Noar S.M., Sutfin E.L. (2019). Systematic Review of Health Communication for Non-Cigarette Tobacco Products. Health Commun..

[11] Dickinson D.M., Johnson S.E., Coleman B.N., Tworek C., Tessman G.K., Alexander J. (2016). The Language of Cigar Use: Focus Group Findings on Cigar Product Terminology. Nicotine Tob. Res..

[12] Trapl E.S., Terchek J.J., Danosky L., Cofie L., Brooks-Russell A., Frank S.H. (2011). Complexity of Measuring “Cigar Use” in Adolescents: Results From a Split Sample Experiment. Nicotine Tob. Res..

[13] Lee S., Cappella J.N., Lerman C., Strasser A.A. (2011). Smoking Cues, Argument Strength, and Perceived Effectiveness of Antismoking PSAs. Nicotine Tob. Res..

[14] Corey C.G., King B.A., Coleman B.N., Delnevo C.D., Husten C.G., Ambrose B.K., Apelberg B.J. (2014). Little Filtered Cigar, Cigarillo, and Premium Cigar Smoking Among Adults—United States, 2012–2013.

[15] Pierce J.P., Choi W.S., Gilpin E.A., Farkas A.J., Merritt R.K. (1996). Validation of Susceptibility as a Predictor of Which Adolescents Take up Smoking in the United States. Health Psychol..

[16] Delnevo C.D., Bover-Manderski M.T., Hrywna M. (2011). Cigar, Marijuana, and Blunt Use among US Adolescents: Are We Accurately Estimating the Prevalence of Cigar Smoking among Youth?. Prev. Med..

[17] Lindblom E.N., Johnson A.C., Gray T., Luta G., Mays D. (2019). How and Why Consumers View “Little Cigars” as Legally-Defined Cigarettes. Tob. Regul. Sci..

[18] Delnevo C.D., Hrywna M., Giovenco D.P., Lo E.J.M., O’Connor R.J. (2017). Close, but No Cigar: Certain Cigars Are Pseudo-Cigarettes Designed to Evade Regulation. Tob. Control.

[19] Huang L.-L., Baker H.M., Meernik C., Ranney L.M., Richardson A., Goldstein A.O. (2017). Impact of Non-Menthol Flavours in Tobacco Products on Perceptions and Use among Youth, Young Adults and Adults: A Systematic Review. Tob. Control.

[20] Maloney E.K., Cappella J.N. (2016). Does Vaping in E-Cigarette Advertisements Affect Tobacco Smoking Urge, Intentions, and Perceptions in Daily, Intermittent, and Former Smokers?. Health Commun..

[21] Sterling K.L., Fryer C.S., Fagan P. (2015). The Most Natural Tobacco Used: A Qualitative Investigation of Young Adult Smokers’ Risk Perceptions of Flavored Little Cigars and Cigarillos. Nicotine Tob. Res..

[22] Stephens M., Ogunsanya M.E., Ford K.H., Bamgbade B.A., Liang M.-C. (2015). Little Cigar and Cigarillo Beliefs and Behaviors among African-American Young Adults. Am. J. Health Behav..

[23] Giovenco D.P., Miller Lo E.J., Lewis M.J., Delnevo C.D. (2017). “They’re Pretty Much Made for Blunts”: Product Features That Facilitate Marijuana Use Among Young Adult Cigarillo Users in the United States. Nicotine Tob. Res..

[24] Trapl E.S., O’Rourke-Suchoff D., Yoder L.D., Cofie L.E., Frank J.L., Fryer C.S. (2017). Youth Acquisition and Situational Use of Cigars, Cigarillos, and Little Cigars: A Cross-Sectional Study. Am. J. Prev. Med..

